# Supplementary material for: Mechanisms governing the pioneering and redistribution capabilities of the non-classical pioneer PU.1
Source: Nat Commun. 2020 Jan 21;11:402. doi: 10.1038/s41467-019-13960-2 (PMC6972792; doi:10.1038/s41467-019-13960-2)
Supplement: Supplementary file 7 — Source data [file 41467_2019_13960_MOESM7_ESM.zip › Source_Data/FigureS2/FigureS2B_MotifScanOutput/KmeansCluster2/homerResults/motif5.similar.html]

motif5

## Information for motif5

T
C
A
G
T
C
A
G
T
C
G
A
C
T
A
G
A
C
T
G
T
C
G
A
C
T
G
A
T
C
A
G
T
C
G
A
T
C
G
A
T
C
A
G
  
Reverse Opposite:  

A
G
T
C
A
G
C
T
A
G
C
T
A
G
T
C
G
A
C
T
A
G
C
T
A
G
T
C
G
A
T
C
A
G
C
T
A
G
T
C
A
G
T
C
  

|  |  |
| --- | --- |
| p-value: | 1e-412 |
| log p-value: | -9.499e+02 |
| Information Content per bp: | 1.647 |
| Number of Target Sequences with motif | 1507.0 |
| Percentage of Target Sequences with motif | 30.00% |
| Number of Background Sequences with motif | 2983.4 |
| Percentage of Background Sequences with motif | 7.34% |
| Average Position of motif in Targets | 101.0 +/- 49.5bp |
| Average Position of motif in Background | 95.3 +/- 100.9bp |
| Strand Bias (log2 ratio + to - strand density) | 0.5 |
| Multiplicity (# of sites on avg that occur together) | 1.58 |
| Motif File: | file (matrix) reverse opposite |

### Similar de novo motifs found

|  |  |  |  |  |  |  |  |
| --- | --- | --- | --- | --- | --- | --- | --- |
| Rank | Match Score | Redundant Motif | P-value | log P-value | % of Targets | % of Background | Motif file |
| 1 | 0.790 | C T A G C G T A T C A G C G T A C G T A T A C G T C G A C T G A A T C G T C G A C G T A A C T G | 1e-246 | -567.358522 | 16.38% | 3.39% | motif file (matrix) |
| 2 | 0.793 | G A C T G A T C G A T C G A C T A G T C G A T C G A C T A G T C G A T C G A C T A G T C G A T C G A C T A G C T | 1e-237 | -545.795109 | 71.23% | 46.95% | motif file (matrix) |
| 3 | 0.768 | A G C T A G T C G A T C G A C T A G T C G A T C G A C T A G T C G A T C G A C T A G T C G A T C G A C T | 1e-108 | -249.562637 | 6.21% | 1.02% | motif file (matrix) |
| 4 | 0.763 | A G T C A C G T A G C T A G T C A C G T A C G T A G T C | 1e-63 | -146.992257 | 31.61% | 20.72% | motif file (matrix) |
